# Immune cell annotation in the single-cell studies: technologies, challenges, and integrative solutions

**DOI:** 10.1007/s12026-026-09780-4

**Published:** 2026-05-01

**Authors:** Sabrina George, Nor Adzimah Johdi

**Affiliations:** UKM Medical Molecular Biology Institute (UMBI), National University Malaysia, Cheras, Malaysia

**Keywords:** Single-cell RNA sequencing, Annotation, Immune cells profiling, Transcriptomic, Proteomic, Multimodal approaches

## Abstract

Single-cell RNA sequencing has transformed immunological research by enabling high-resolution transcriptional profiling of individual immune cells. Despite its transformative impact, annotating immune cells based solely on transcriptomic data remains challenging. These difficulties arise from biological factors, including gene expression heterogeneity and post-transcriptional regulation, as well as technical limitations that contribute to mismatches between mRNA and protein expression. Such discrepancies may lead to cell misclassification and obscure functional insights, particularly in heterogeneous populations such as peripheral blood mononuclear cells. This review highlights the major challenges in immune cell annotation by detailing the mechanisms underlying mRNA-protein discrepancies, examining both the biological factors and technical artifacts that drive this divergence, and emphasizing their implications for accurate cell classification. A critical overview of current single-cell profiling technologies follows, with evaluation of the respective advantages and limitations of transcriptomic, proteomic, and multimodal approaches. In particular, technologies such as Cellular Indexing of Transcriptomes and Epitopes by Sequencing integrate transcriptomic and proteomic data, addressing the shortcomings of single-modality analyses. Further examination focuses on computational strategies for immune cell annotation, with emphasis on automated methods and bioinformatics frameworks tailored to multi-omics datasets. The unique computational challenges of integrating mRNA and protein data, together with solutions for improved annotation accuracy, are discussed. This review integrates key challenges, technologies, and computational tools, highlighting the need for standardized multimodal profiling of immune cells. Such integration enhances annotation reliability and advances disease understanding and therapy discovery.

## Introduction

Single-cell RNA sequencing (scRNA-seq) has revolutionized the field of immunology by enabling detailed transcriptional profiling at single-cell resolution. This technology allows researchers to dissect immune cell heterogeneity, trace developmental trajectories, and study dynamic immune responses in both health and disease [1, 2]. However, one of the primary limitations of scRNA-seq is its reliance on transcriptomic data alone for cell-type annotation. This poses particular challenges in studies of peripheral blood mononuclear cells (PBMCs), where closely related immune subsets often exhibit overlapping gene expression profiles [3, 4]. Accurate annotation is essential for identifying immune responses, uncovering disease-associated signatures, and guiding therapeutic interventions. For example, precise classification of monocyte subsets in COVID-19 and influenza has revealed immune signatures linked to disease severity and treatment targets [5].

A key obstacle to accurately annotating immune cells is the discordance between transcript and protein abundance. Because immune-cell identity and function are chiefly governed at the protein stage via post-transcriptional regulation, translational control, and protein degradation [6], mRNA readouts can diverge markedly from the corresponding protein expression, leading to misclassification of cell types [7]. For instance, CD15 surface marker, encoded by the gene *FUT4*, is highly expressed on myeloid-derived suppressor cells (MDSCs), yet its mRNA is often undetectable in scRNA-seq, highlighting how transcript-based annotation may fail to capture immunosuppressive populations that are clearly defined at the protein level [6]. Similarly, immune checkpoint protein PD-1, encoded by *PDCD1*, may be abundantly expressed on the surface of exhausted T cells while showing minimal mRNA expression, resulting in the underestimation of immunoregulatory states when relying solely on transcriptomic data [8].

This discordance is further compounded by technical limitations inherent to scRNA-seq. For example, dropout events, caused by low capture efficiency or stochastic gene expression, can result in undetected transcripts, especially those expressed at low abundance [9]. Additionally, transcripts with short half-lives may degrade rapidly, leading to insufficient mRNA for sequencing [3]. Furthermore, shallow sequencing depth further exacerbates these issues by reducing the likelihood of detecting key markers essential for accurate cell-type identification [10]. Collectively, these factors impair the sensitivity of mRNAs encoding critical surface proteins, such as CD25, CD69, or PD-1, which are central to defining T cell activation and immune regulation.

Traditionally, immune cell annotation has been based on surface protein expression, with flow cytometry remaining the gold standard for immunophenotyping. Protein-based methods enable precise classification using well-characterized markers such as CD3, CD4, CD14, PD-1, and HLA-DR, and have been pivotal in studies of hematopoiesis and tumor immunology [11, 12]. However, these approaches lack of transcriptomic resolution and are limited in multiplexing capacity. The development of multimodal technologies such as cellular indexing of transcriptomes and epitopes by sequencing (CITE-seq) has bridged this gap, enabling the simultaneous measurement of gene expression and surface protein expression in single cells. Despite their potential, widespread adoption of such technologies remains limited due to the technical barriers and costs involved.

Accurate immune cell annotation does not depend on a single data modality, but rather emerges from the interaction between biological regulation, technological measurement, and computational interpretation. In practice, annotation accuracy is shaped by three interdependent factors: (i) biological mechanisms governing the relationship between mRNA and protein expression, (ii) technological constraints that determine which cellular features can be reliably captured, and (iii) computational frameworks used to integrate and interpret heterogeneous single-cell data. Failure to account for any one of these dimensions can lead to incomplete or misleading immune cell classification.

This mini-review explores the challenges of immune cell annotation in single-cell studies through this integrated lens. We first outline the key biological and technical challenges underlying immune cell annotation, with particular emphasis on mRNA-protein discordance. We then review transcriptomic, proteomic, spatial, and multimodal technologies in terms of how they enable or constrain immune cell annotation, rather than as standalone platforms. Finally, we discuss computational approaches for integrating multimodal data and highlight unresolved challenges and future directions toward more robust and biologically grounded immune cell annotation strategies.

## Importance and key challenges in immune cell annotation

Accurate immune cell annotation is fundamental to single-cell transcriptomics and essential for uncovering biological insights in immunology. In the context of cancer, infection, and autoimmune diseases, the ability to reliably identify and characterize immune cell types and states is crucial for mapping cellular heterogeneity, tracing differentiation trajectories, and understanding disease progression at single-cell resolution. As scRNA-seq and multimodal omics technologies evolve, the demand for precise annotation has become increasingly vital for both basic research and clinical applications [13, 14].

In practice, immune cell annotation follows a structured analytical pipeline that integrates biological interpretation with technical preprocessing. Single-cell data are first generated using transcriptomic, proteomic, or multimodal platforms, followed by stringent quality control to remove low-quality cells and technical artifacts. Normalization and batch correction are then applied to mitigate technical variability across samples and experimental conditions. Dimensionality reduction and clustering enable the identification of transcriptionally or phenotypically similar cell populations, which are subsequently annotated using marker-based, reference-based, or integrative computational approaches. At each stage, biological regulation and technical constraints jointly influence annotation accuracy, underscoring the importance of considering mRNA–protein relationships and platform-specific limitations when interpreting immune cell identities.

Cell annotation transforms raw sequencing data into biologically interpretable information. By assigning cellular identities based on molecular signatures, researchers can deconstruct complex immune landscapes and identify rare or transitional populations such as tissue-resident memory T cells (Trm), regulatory dendritic cells, and T peripheral helper (Tph) cells [15, 16]. Without accurate annotation, such populations may be misclassified or overlooked, obscuring their functional relevance in immune surveillance, regulation, or tumor suppression.

Despite its importance, immune cell annotation from scRNA-seq data remains inherently challenging due to substantial gene expression heterogeneity, technical noise, and sparsity [17]. These issues are especially pronounced in immune populations, where closely related cell types often share overlapping transcriptional profiles. For instance, both CD8 + cytotoxic T cells and natural killer (NK) cells express granzymes (*GZMB*) and perforin (*PRF1*), making it difficult to distinguish between them using transcriptomic data alone [18]. Similarly, transitional stages between B cells and plasma cells can display ambiguous expression patterns, with both expressing immunoglobulin genes [19]. Further complexity arises in dynamic states such as activation, exhaustion, or tissue residency, where conventional markers such as *PDCD1* and *CD39* are expressed at low or fluctuating levels, complicating annotation [20, 21].

The most common annotation strategy involves unsupervised clustering via dimensionality reduction techniques such as t-distributed stochastic neighbour embedding (t-SNE) or uniform manifold approximation and projection (UMAP), followed by manual inspection of canonical marker gene expression. While widely adopted, this approach is time-consuming, subjective, and lacks scalability for large datasets. Critically, it risks misclassifying or missing rare and functionally distinct populations, particularly when marker genes are absent, downregulated, or when mRNA and protein expression are discordant. These limitations are especially evident in disease contexts. In the context of COVID-19, precise delineation of monocyte and dendritic cell subsets was crucial for correlating immune responses with disease severity and patient outcomes [22]. In cancer immunology, poor annotation may lead to underestimation of immunosuppressive populations such as myeloid-derived suppressor cells (MDSCs), which often express low levels of lineage-specific transcripts, or to the misclassification of exhausted T cells when checkpoint receptor transcripts are absent [23].

Transcript–protein discordance represents a major barrier to accurate cell annotation. The mRNA levels do not always correlate with surface protein abundance due to post-transcriptional regulation, protein degradation, and translational control (Liu et al., 2016). For instance, exhausted T cells frequently exhibit high surface expression of immune checkpoint proteins such as CTLA-4, even when their corresponding transcripts, CTLA-4, are undetectable in scRNA-seq data. This discrepancy can lead to the misclassification of these cells as active effector populations [24]. The observed mRNA-protein discordance likely stems from tight post-transcriptional regulation, where checkpoint-encoding transcripts undergo rapid degradation following translation, resulting in transient mRNA visibility despite sustained protein expression [25]. Thus, the integration of surface protein data via multimodal approaches like CITE-seq and RNA Expression and Protein sequencing (REAP-seq) has proven instrumental in mitigating mRNA-protein discordance. These techniques employ DNA-barcoded antibodies to simultaneously quantify transcripts and surface proteins in single cells, enabling direct detection of stable surface markers, even when their cognate mRNAs are absent or degraded [26, 27].

As summarized in Table 1, current computational tools have significantly advanced automated immune cell annotation, yet their generalizability across disease contexts remains a topic of active investigation. While methods like Collaborative Agent System for Single-cell Interpretable Annotation (CASSIA) and Area Under the Curve (AUC) cell-based approaches show improved performance, persistent limitations, including difficulty in annotating novel populations, capturing transitional states, and ensuring cross-platform reproducibility, still emerge as common challenges across these tools (Table 1). These gaps highlight the need for next-generation annotation frameworks that integrate multimodal data such as transcriptomic, proteomic, and epigenomic layers, which represent a promising avenue but will require rigorous benchmarking and biological validation to ensure both interpretability and cross-study consistency.

The consequences of poor annotation are far-reaching, with misclassification potentially distorting biological interpretations, misguiding therapeutic predictions, and obscuring disease mechanisms. For instance, mislabelling exhausted T cells as activated effectors could lead to erroneous predictions of patient responses to immune checkpoint inhibitors [23]. Similarly, failure to detect tissue-resident macrophages due to weak transcript signals may underestimate their contributions to inflammation or tissue remodelling. Inconsistent annotation practices further impede the development of reliable immune atlases and complicate cross-study meta-analyses [28]. To address these challenges, initiatives like the Human Cell Atlas are advancing standardized guidelines, curated reference datasets, and benchmarking protocols to enhance reproducibility and data harmonization [29].

Collectively, immune cell annotation is not merely a technical step but a critical determinant of rigor in single-cell immunology. Emerging multimodal technologies, coupled with improved computational tools and community-driven reference frameworks, now empower researchers to decipher complex immune landscapes with unprecedented resolution.

**Table 1:** Computational tools for automated immune cell annotation

Tool	Strengths	Limitations	Reference
ImmunIC	High accuracy of up to 98% for major immune lineages	Limited resolution for fine-grained subtypes	[17]
ImmClassifier	Improved distinction of nuanced subsets (e.g., CD8 + effector memory T cells)	Limited generalizability to novel/intermediate states, as it is trained on reference datasets	[30]
sc-ImmuCC	Uses a hierarchical framework which achieves 71–90% accuracy	Struggles with rare subsets (e.g., γδ T cells, MAIT cells)	[5]
CASSIA	Superior performance (> 25% improvement over others); excels in functionally relevant states	Not explicitly discussed, as it may require benchmarking for novel states	[31]
AUCell-based method	Consistently outperforms Azimuth; automated label assignment using marker gene sets	Reliance on predefined marker lists limits its adaptability when novel or context-specific markers emerge.	[32]
Garnett	Robust and accurate when classifying immune cells even with rare or missing cell types, and when data quality is low.	Limitation in identifying novel cells that are not included in the original marker file	[33]
scBalance	Scales effectively to million-cell datasets and offer a fast and stable computation speeds	Relies on quality reference atlases, potentially struggling with novel or highly divergent immune cell states	[34]
TCAT	Derives a catalogue of gene expression programs (GEPs) from multiple T-cell scRNA-seq references spanning tissues	Reliance on predefined GEPs, potentially missing novel states, and focus primarily on T-cells rather than broad immune annotation	[35]
CellTypist	Provides fast annotation using curated references, achieving high accuracy for immune cell subtypes like naïve/memory T/B cells	Struggles distinguishing fine-grained states such as naive vs. central memory T-cells without CITE-seq	[36]
Symphony	Robustly handling noisy or incomplete labels in immune scRNA-seq while preserving batch-corrected structures	High computational demands for training on large references and potential overfitting risks	[37]
TACIT	Uncovers rare populations effectively in spatial/multi-omics contexts adaptable to scRNA-seq markers	Needs for abundant background signals, risk of unassigned cells from poor intensities or novel types, and unsuitability for small cell regions	[38]

Abbreviation: *ImmunIC* Immune Cell Identifier and Classifier, *MAIT* Mucosal-associated invariant T, *CASSIA* Collective Agent System for Single-Cell Interpretable Annotation, *TCAT* T-CellAnnoTator, *TACIT* Threshold-based Assignment of Cell Types from Multiplexed Imaging DaTa

## Mechanisms underlying mrna-protein discrepancies in immune cells

The correlation between mRNA abundance and protein expression is a central tenet in molecular biology, yet this relationship is frequently weak or inconsistent in immune cells, a phenomenon with significant implications for both immunological research and clinical applications. While mRNA provides the transcriptional template for protein synthesis, it is the proteins themselves that mediate critical immune functions, including antigen presentation, cytokine signalling, and cytotoxic activity. Given the dynamic nature and tight regulation of immune responses, understanding the discordance between transcript and protein levels is essential for accurate immune cell characterization and functional annotation [39, 40]. This ensures coverage of the full spectrum of immune responses, capturing phenotypic and functional diversity, reducing cell-state misclassification, and advancing immunological insight.

Although a direct, linear mRNA-protein relationship is often assumed, extensive empirical evidence contradicts this presumption [41, 42]. For instance, key immune mediators such as interferons (IFN-α), tumor necrosis factor (TNF) receptor superfamily agonists, and chemokines routinely exhibit non-linear transcript-protein relationships due to stringent post-transcriptional and translational control [43]. This is particularly apparent in the melanoma microenvironment, where distinct immune subtypes display divergent expression patterns of checkpoint proteins and cytokine regulators such as CXCL10 and IFNAR1 [44]. These disparities directly influence immune phenotypes and therapeutic responses, including outcomes to mRNA-based vaccines.

Such discordances arise from interconnected biological and technical factors, reflecting the multilayered regulation spanning transcription to functional protein production [7]. In immune cells, where rapid post-transcriptional and protein turnover are essential for responding to environmental stimuli, these disconnects are particularly pronounced. Consequently, elucidating the mechanisms underlying mRNA-protein uncoupling not only refines the interpretation of single-cell multiomics data but also provides fundamental insights into immune physiology and pathology.

### Biological factors influencing mRNA-protein expression

**Fig. 1:**
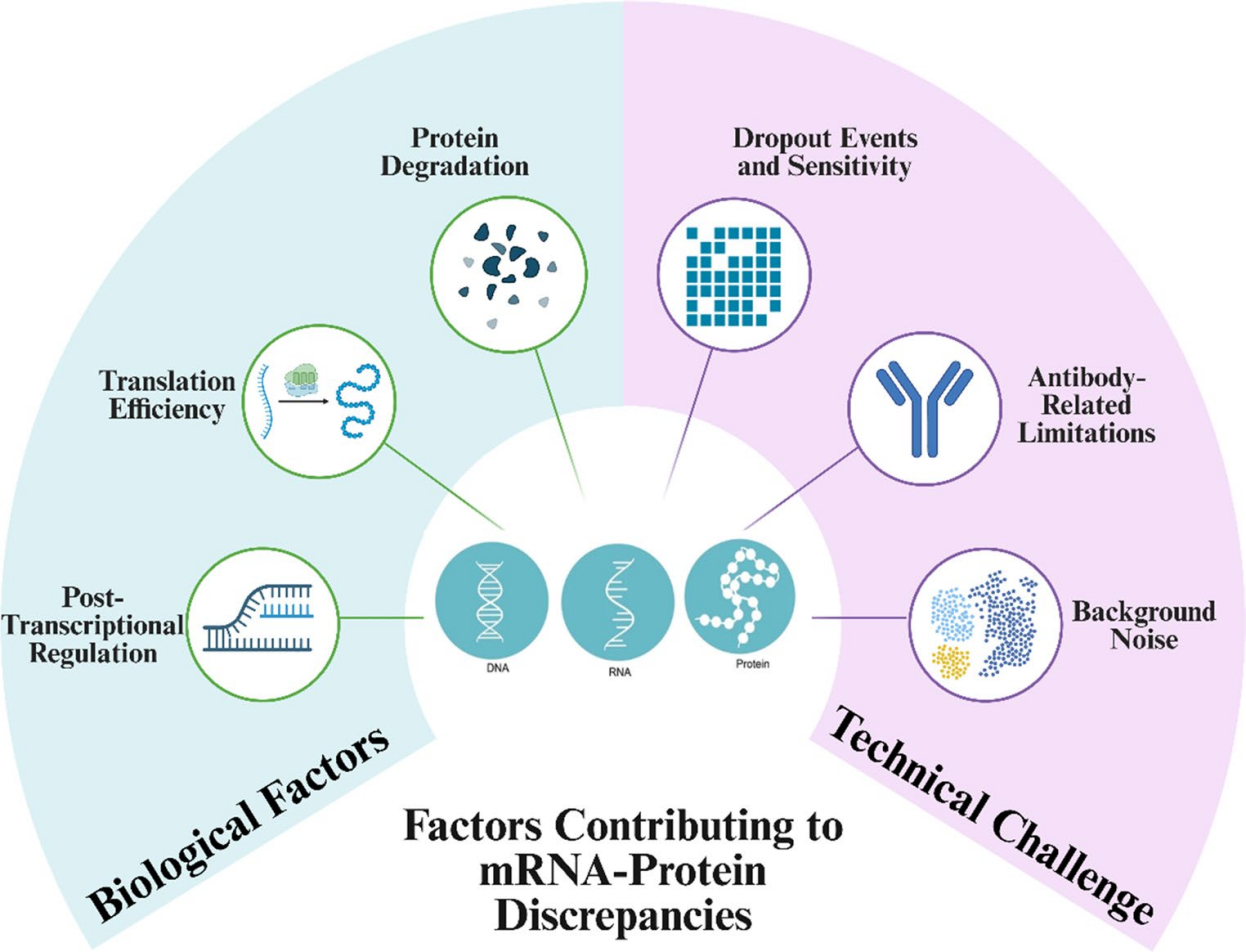
Biological and technical factors contributing to mRNA-protein discordance in a single-cell study. Illustration created using BioRender (https://biorender.com)

The disparity between mRNA and protein levels in immune cells is governed by three key biological processes, which are post-transcriptional regulation, translation efficiency, and protein degradation (Fig. 1) [45–47]. These mechanisms operate in a cell-type and context-specific manner, with particular relevance in immune cells due to their need for rapid functional shifts during immune surveillance and activation.

Post-transcriptional mechanisms significantly influence transcript stability and translational capacity [48]. Central to this process are RNA-binding proteins (RBPs) and microRNAs (miRNAs), which modulate mRNA degradation or inhibit translation, thereby decoupling transcript levels from protein synthesis [49, 50]. In immune cells such as macrophages and T cells, inflammatory cytokine mRNAs such as IL-6 and TNF are tightly controlled by RBPs, including tristetraprolin (TTP), Regnase-1, AUF1, and Roquin [51]. These regulators bind to AU-rich elements (AREs) in the 3’ UTRs of target mRNAs, destabilizing transcripts or suppressing their translation. This fine-tuning is crucial for preventing excessive cytokine production during immune activation while still enabling rapid responses. For example, TLR-stimulated macrophages transiently transcribe cytokine mRNAs, but their short half-lives, which are dictated by RBP-mediated decay, ensure tight control over protein output, independent of transcriptional bursts [52].

Even when mRNA is abundant, its conversion into protein depends heavily on translation efficiency, which varies based on multiple factors such as ribosome binding site (RBS), codon composition, tRNA availability, and ribosomal dynamics [53, 54]. A strong RBS enhances ribosome recruitment and increases translation initiation, while optimal codon usage increases translation speed [55]. Additionally, the initiation and elongation phases of translation are modulated by various cis-regulatory elements. For example, sequences such as polyproline motifs can cause ribosome stalling during elongation, reducing overall protein output [56]. In immune cells, where rapid protein production is essential, these bottlenecks significantly impact functional output. If elongation stalls exceed initiation rates, protein synthesis is drastically impaired [57, 58].

Finally, protein abundance is dynamically regulated by degradation pathways, which operate independently of mRNA turnover [59]. Key mechanisms include the ubiquitin-proteasome system, lysosomal autophagy, and non-specific proteolysis [60–62]. These pathways selectively eliminate damaged, misfolded, or excess proteins and can contribute to reduced protein levels even in the presence of abundant transcripts. Interestingly, studies have demonstrated that mRNA and protein degradation can be uncoupled. In prostate tissue, mRNA turnover occurs at a high rate, yet protein stability remains unaffected, suggesting these processes are independently regulated [63, 64]. Moreover, many proteins exhibit longer half-lives than their corresponding transcripts, allowing for sustained protein function despite transcriptional downregulation. This temporal buffering is particularly relevant in central and effector memory T cells [25], macrophage subsets [65], and exhausted T cell [66] where persistent protein expression is needed for prolonged signalling or effector functions.

### Technical factors influencing mRNA-protein expression

In addition to biological regulation, various technical factors inherent to single cell multiomics platforms significantly affect the observed correlation between mRNA and protein expression. These include issues such as dropout events, antibody-related limitations, and background noise, all of which can distort true cellular expression profiles and complicate data interpretation (Fig. 1).

A primary technical challenge in scRNA-seq is the occurrence of dropout events, where mRNA transcripts fail to be detected despite their presence in the cell [67]. These events stem from both the stochastic nature of gene expression and technical limitations such as inefficient mRNA capture and reverse transcription, particularly for low-abundance transcripts [68]. Dropouts disproportionately affect genes with transient or low expression, including critical immune regulatory genes activated under specific stimuli or stress conditions [67]. As a result, the incomplete detection of these transcripts can lead to misinterpretation of cellular states and obscure the detection of key functional pathways. To address this, various imputation algorithms have been developed, each employing distinct computational strategies.

These include DrImpute, which utilizes a clustering-based approach that aggregates similar cells to improve local expression patterns, making it particularly effective for enhancing cell type clustering [69]. In contrast, scDoc implements a probabilistic model that specifically distinguishes biological absence of expression from technical dropouts, providing more accurate recovery of true zeros in low-coverage datasets [68]. The scHinter algorithm takes a different approach by applying hierarchical network regularization to maintain gene-cell relationships during imputation, offering robust performance for noisy or sparse data [70].

More advanced methods like I-Impute integrate multiple data modalities by incorporating biological prior knowledge alongside expression data, enabling context-aware imputation, which is particularly valuable for immune cell states [71]. Most recently, CCI employs deep learning through diffusion models to capture complex global expression patterns, showing superior performance for challenging datasets such as those capturing immune cell activation dynamics [72]. While these tools have significantly improved the resolution of single-cell transcriptomic analyses, dropout-related inaccuracies still impact downstream interpretation. The choice of algorithm depends heavily on experimental context, where DrImpute may suffice for basic cell type identification, CCI’s sophisticated pattern recognition becomes crucial when analyzing dynamic immune responses. All methods nevertheless share inherent limitations in completely resolving dropout artifacts, which can still lead to biased biological interpretations in tasks like differential expression analysis and cell state annotation.

In multimodal single-cell technologies, antibody-based detection techniques such as CITE-seq, REAP-seq and AbSeq enable simultaneous profiling of surface protein expression alongside transcriptomic data. These techniques are widely implemented on platforms like BD Rhapsody™ and 10x Genomics™ Chromium, facilitating integrated multi-omics analysis at single-cell resolution. However, several antibody-related issues may introduce artifacts that compromise the accuracy of mRNA–protein correlations. One critical limitation is antibody specificity. Off-target binding or failure to distinguish between closely related protein isoforms can result in false-positive or ambiguous signals [73]. In complex immune environments, where closely related proteins are often co-expressed, cross-reactivity poses a significant challenge, leading to non-specific binding and distorted protein measurements [74].

Additionally, antibody affinity and saturation further complicate the protein quantification. At high antigen concentrations, antibody saturation can occur, limiting the dynamic range of detection and potentially masking subtle but biologically meaningful differences [75]. Conversely, low-affinity antibodies may fail to detect proteins present at low abundance. A more fundamental constraint is the limited availability of well-validated antibodies for many immune targets. Commercially available antibodies often lack comprehensive validation for single-cell applications, particularly for emerging immune markers or unconventional protein isoforms. To mitigate these effects, researchers must employ rigorous antibody validation, panel optimization, and high-affinity reagents when available. However, even these measures are constrained by the commercial antibody landscape. Alternative solutions include custom antibody development, though this is time-consuming and costly, epitope tagging strategies for recombinant proteins, and computational correction methods to account for antibody noise.

Another major technical challenge is background noise, particularly in droplet-based platforms such as 10x Genomics™ Chromium systems. Protein measurements via DNA-barcoded antibodies (CITE-seq) are prone to contamination from unbound antibody carryover, non-specific binding, and ambient mRNA/protein in empty droplets [76]. In some cases, antibody-derived UMIs in empty droplets even exceeded those in droplets containing cells, emphasizing the magnitude of the ambient signal [77]. This necessitates normalization approaches such as “dsb” (denoised and scaled by background), which leverage empty droplet profiles for denoising. Comparative analyses of 10x Chromium and BD Rhapsody™ further highlight platform-specific noise profiles in which BD Rhapsody™ tends to display a more uniform but generally higher baseline of ambient noise, while 10x Chromium™ shows lower overall background but exhibits stronger cell type–specific biases, particularly from neutrophil-derived transcripts in damaged samples [78]. These differences, rooted in the platforms’ distinct molecular designs—microwell versus droplet systems, highlight the importance of computational correction tools such as dsb for reliable immune cell profiling. Finally, the major biological and technical sources of mRNA–protein discordance and their implications for immune cell annotation are summarized in Table 2.

**Table 2:** Biological and technical sources of mRNA–protein discordance and their implications for immune cell annotation

Source of mRNA–protein discordance	Implications for immune cell annotation
Post-transcriptional regulation (e.g. miRNA control, RNA stability)	Protein-defined immune states may be underrepresented or misclassified when relying on transcript-based markers
Translational control and protein stability	Functional immune phenotypes can persist despite low or transient mRNA expression, leading to annotation bias
Transcript dropout in scRNA-seq	False-negative marker detection increases annotation uncertainty and ambiguous cell identities
Limited sequencing depth	Low-abundance marker genes essential for immune subset definition may be missed
Stochastic capture efficiency	Inconsistent marker detection across cells reduces annotation robustness and reproducibility

## Technologies for immune cell profiling

Advances in transcriptomic and proteomic technologies have fundamentally reshaped our understanding of immune cell diversity, function, and regulation. Early immune profiling methods relied heavily on microarrays, which enabled gene expression analysis across predefined probes [79]. While instrumental in biomarker discovery and disease stratification, microarrays were limited by probe dependency, hybridization artifacts, narrow dynamic range, and high RNA input requirements, rendering them suboptimal for analyzing rare or low-yield immune samples [80, 81]. The advent of bulk RNA sequencing (RNA-seq) overcame these limitations by enabling transcriptome-wide, probe-independent detection of gene expression with enhanced sensitivity and dynamic range [82–85]. This approach uncovered novel transcripts, alternative splicing events, and non-coding RNAs. However, bulk RNA-seq averages gene expression across mixed populations, thereby masking cellular heterogeneity and limiting its utility in resolving rare or transient immune states [86].

The introduction of scRNA-seq marked a transformative step, enabling transcriptomic profiling at single-cell resolution. Unlike bulk RNA-seq, which averages signals across cell populations, scRNA-seq isolates individual cells via microfluidics, droplet-based encapsulation, or microwell plates. The individual cells are then lysed, and their RNA molecules are uniquely barcoded during reverse transcription. By quantifying gene expression per cell, this technology has revealed rare subsets, delineated developmental trajectories, and captured context-specific immune responses in cancer, infection, and autoimmunity that are otherwise obscured in bulk analyses [87–89]. Nonetheless, scRNA-seq poses challenges, including loss of spatial context, variable capture efficiency, and limited transcript recovery, particularly from small immune cells. These limitations are addressed by spatial transcriptomics technologies such as Multiplexed Error-Robust Fluorescence In Situ Hybridization (MERFISH) and Sequential Fluorescence In Situ Hybridization (seqFISH), which preserve tissue architecture while mapping transcripts in situ [90, 91].

MERFISH employs combinatorial binary barcoding and sequential imaging to detect hundreds to tens of thousands of genes simultaneously with nanometre-scale spatial localization, offering robust error correction quantification of preselected gene panels [90]. In contrast, seqFISH uses color-coded barcodes combined over multiple cycles to achieve super-resolved imaging, excelling in detecting low-copy transcripts like transcription factors [91]. Both methods surpass scRNA-seq in spatial mapping and sensitivity for targeted genes, including lower dropout than scRNA-seq but are restricted to predefined gene sets. Complementing these approaches, single-cell ATAC-seq (scATAC-seq) profiles chromatin accessibility at the single-cell level using a hyperactive Tn5 transposase to insert sequencing adapters into open chromatin regions [92]. This reveals active regulatory elements like enhancers or promoters, and infers transcription factor binding, thereby linking epigenetic states to immune cell function. When integrated with scRNA-seq, scATAC-seq provides a multi-omics framework to decipher gene regulatory mechanisms underlying immune responses.

While transcriptomics provides information on gene activity, proteomic technologies are essential for functional immune profiling, since proteins are the effectors of immune responses. Flow cytometry has long served as the cornerstone for immunophenotyping, allowing multiparametric quantification of surface and intracellular markers, functional states, and cytokine production [93, 94]. Clinically, it has been extensively used to validate immune signatures in cancer, autoimmune diseases, and infectious conditions such as COVID-19 [95, 96]. However, conventional flow cytometry is constrained by spectral overlap (typically limiting multiplexing to ~ 15–20 markers), the need for meticulous spillover compensation of antibody–fluorophore conjugates, laser-line restrictions on concurrent excitation, and diminishing signal-to-noise in larger panels. Consequently, Spectral flow cytometry partially overcomes these constraints by capturing full emission spectra rather than discrete channels, enabling simultaneous detection of 30–50 markers with reduced compensation artifacts [97, 98]. This approach enhances resolution for dim markers in cytokines and improves the unmixing of fluorophores with overlapping spectra. However, spectral systems demand extensive pre-panel optimization, reference controls for spectral unmixing, and higher computational costs for data deconvolution. Additionally, they remain constrained by antibody quality and cellular autofluorescence in certain types of samples.

Mass cytometry (CyTOF) further extended immune profiling capabilities by using metal-conjugated antibodies and time-of-flight mass spectrometry, enabling simultaneous detection of over 40 proteins per cell without spectral interference [99]. The CyTOF has been instrumental in identifying rare immune populations and intracellular signalling events in cancer and autoimmunity, although it lacks spatial resolution and requires specialized instrumentation [100, 101]. Thus, Imaging mass cytometry (IMC) overcomes CyTOF’s spatial limitations by combining high-dimensional proteomics with tissue imaging. In IMC, tissue sections stained with metal-tagged antibodies are ablated pixel-by-pixel using a laser, and the vaporized metal ions are quantified by mass spectrometry [102, 103]. This preserves tissue architecture while enabling multiplexed detection of more than 40 markers, revealing immune-tumor interactions, tissue-resident niches, and cellular neighborhoods in situ. However, IMC suffers from lower throughput, complex data analysis pipelines, and higher costs compared to suspension CyTOF.

Thus, the growing complexity of immune responses has driven innovation in multimodal profiling platforms. Building on their ability to resolve mRNA-protein discrepancies, as discussed earlier, CITE-seq and REAP-seq have become particularly valuable for identifying immune subsets where protein expression diverges from transcriptional profiles. For example, they are reliable in detecting CD103 + tissue-resident memory T cells and FCεRIα + mast cells that frequently escape transcriptomic identification [104]. Furthermore, these methods enable more accurate discrimination of functionally distinct states, such as differentiating activated (CD69 + CD25+) from exhausted (PD-1 + TIM-3+) T cell populations within tumor microenvironments [105].

Building upon the CITE-seq multimodal framework, Extended CITE-seq (ECCITE-seq), an advanced version of the original CITE-seq, significantly expands single-cell profiling capabilities by integrating four key modalities, which are transcriptome-wide mRNA sequencing, surface protein quantification via antibody-derived tags (ADTs), paired T-cell receptor/B-cell receptor (TCR/BCR) sequencing for immune clonotype tracking, and detection of CRISPR single-guide RNAs (sgRNAs) for perturbation screening [106]. Unlike conventional CITE-seq or REAP-seq which combine only transcriptomics and proteomics, ECCITE-seq’s unique value lies in its ability to simultaneously capture genetic perturbations, via sgRNA detection, while maintaining immune receptive specificity. Therefore, making it particularly powerful for functional immunology studies investigating gene knockout effects on immune cell phenotypes [106, 107].

While multimodal approaches such as ECCITE-seq provide high-dimensional mRNA–protein profiling, they lack spatial context, which is critical for immune cell annotation in tissues where cellular identity and function depend on local microenvironmental cues. Spatial transcriptomic platforms address this limitation by preserving tissue architecture, cellular morphology, and spatial co-localization, thereby enabling immune cell annotation that integrates molecular identity with positional information.

Spatial approaches such as CosMx^®^, Visium, and Xenium improve immune cell annotation by resolving immune populations within their native tissue niches, facilitating discrimination of transcriptionally similar but spatially distinct immune states. This is particularly relevant for immune subsets such as tumor-associated macrophages (TAMs) [108], tissue-resident myeloid cells, and neutrophils, which are often underrepresented or ambiguously annotated in dissociation-based scRNA-seq due to transcriptional sparsity or loss during tissue processing. Among spatial platforms, Visium supports discovery-driven annotation through transcriptome-wide spatial mapping integrated with histological context, albeit at multi-cellular resolution [109], whereas Xenium and CosMx^®^ enable higher-resolution, targeted spatial profiling with the capacity to integrate RNA and protein signals [110]. Such multimodal spatial integration enhances annotation robustness by cross-validating immune identities across molecular expression, morphology, and spatial co-localization, allowing more confident resolution of immune cell states within complex tissue microenvironments.

For studies prioritizing throughput over spatial context, the Chromium GEM-X platform (10x Genomics™), an advanced generation of droplet-based scRNA-seq, leverages microfluidics and optimized GEM-X chemistry to achieve high-throughput single-cell multiomics [111]. Its gel bead-in-emulsion (GEM) system supports simultaneous transcriptome-wide RNA-seq, surface protein detection via ADTs, and multiplexed analysis of up to 128 samples per run, making it well-suited for large-scale cohort studies [112]. Nonetheless, GEM-X shares common drawbacks of scRNA-seq platforms, including the absence of spatial information, high costs for expansive cohorts, and reduced sensitivity for rare immune subsets.

To address these limitations, emerging technologies like Evercode™ Immune Profiling (Parse Biosciences) employ a microfluidics-free, combinatorial indexing approach that enables ultra-high-throughput analysis of up to 1 million fixed cells per experiment, which is a 10-fold increase over Chromium GEM-X’s typical throughput. Unlike Chromium’s droplet-based GEM system, Evercode™ utilizes split-pool barcoding to progressively tag transcripts and paired immune receptors across multiple rounds of indexing, eliminating the need for specialized instrumentation [113, 114]. This approach offers a deep clonotype resolution and full-length immune receptor sequencing with matched transcriptomes. However, Evercode™ currently lacks Chromium GEM-X’s integrated protein detection capability. Where Chromium excels in multimodal profiling with standardized workflows, Evercode™ prioritizes massive-scale immune repertoire studies, making them complementary rather than competing solutions.

More recently, Beacon Discovery™ (Bruker) introduced an optofluidic platform designed for real-time, functional single-cell analysis, ideal for immunology, oncology, and therapeutic discovery. The system utilizes Opto-Electrical Positioning (OEP) paired with OptoSelect™ microfluidic chips, where light-actuated pixels precisely isolate live cells into NanoPen chambers for dynamic profiling. Unlike Evercode’s sequencing focus or Chromium GEM-X’s high-throughput omics, Beacon platform prioritizes functional immune metrics, which is demonstrated in adoptive T-cell therapy studies where it quantified single-cell cytotoxicity via caspase-3 activation, cytokine secretion, and phenotype switching during immune synapse formation [115]. These different techniques are summarized in Table 3. 

**Table 3:** Conceptual overview of single-cell and spatial platforms and their implications for immune cell annotation

Conceptual Category	Platform Examples	Data Type	Key Advantages for Immune Annotation	Key Limitations for Immune Annotation
Transcriptomic	scRNA-seq	mRNA	Enables discovery of immune cell states and transcriptional programs	Limited sensitivity for surface markers; dropout affects annotation
Spatial transcriptomic	Visium, Xenium, CosMx	Spatial mRNA (± protein)	Preserves tissue context and cell–cell interactions; improves annotation of spatially defined immune populations	Limited gene panels or resolution constraints depending on platform
Proteomic	Flow cytometry, CyTOF, IMC	Surface/intracellular proteins	High sensitivity for canonical immune markers; robust phenotyping	Restricted to predefined antibody panels; limited discovery potential
Multimodal	CITE-seq, ECCITE-seq	mRNA + protein	Improves annotation confidence by integrating transcriptomic and proteomic signals	Antibody-dependent; increased analytical complexity

Abbreviation: *scRNA-seq* Single-cell RNA sequencing, *CyTOF* Cytometry by Time-of-Flight (Mass Cytometry), *IMC* Imaging Mass Cytometry, *CITE-seq* Cellular Indexing of Transcriptomes and Epitopes by sequencing, *ECCITE-seq* Expanded CRISPR-compatible Cellular Indexing of Transcriptomes and Epitopes

## Bioinformatic obstacles in integrating mrna and protein datasets

The field of single-cell multimodal analysis has evolved significantly since the introduction of pioneering technologies like CITE-seq and REAP-seq, which first enabled simultaneous measurement of mRNA and protein expression within individual cells [26, 27]. These methods have been widely implemented on high-throughput platforms such as 10x Genomics™ Chromium and BD Rhapsody™, establishing them as standard workflows in immunology research. Modern iterations like 10x Genomics™ Chromium and BD Rhapsody™ now support even larger-scale multimodal profiling while addressing early limitations in sensitivity and throughput [89, 116]. Despite these technological advances, computational integration of mRNA and protein datasets remains challenging due to fundamental differences in the data structure of mRNA and protein.

One of the primary barriers to effective integration is the disparity in data sparsity and distribution between transcriptomic and proteomic measurements. The scRNA-seq generates high-dimensional count matrices exhibiting extreme sparsity, with 60–90% zero values per cell due to transcriptional stochasticity and technical dropout events [117]. This contrasts sharply with bulk RNA-seq, where the proportion of zeros typically ranges between 10% and 40%, necessitating distinct statistical modelling approaches. While scRNA-seq requires zero-inflated or negative binomial distributions to account for this sparsity, ADT-based protein data, although less sparse, are constrained by smaller panel sizes of typically 30–200 markers [118] and confounded by technical artifacts, including non-specific binding, cross-reactivity, and antibody saturation [119, 120]. As a result, normalization approaches developed for transcriptomic data, such as log-transformation or library size scaling, are often inappropriate for protein data, which may require distinct strategies for batch correction and scaling. Moreover, differences in the dynamic range and detection sensitivity between RNA and protein further complicate joint analysis. Specifically, RNA data require normalization methods that address high variability and dropout rates, as previously mentioned, while protein data, with their broader dynamic range and more stable signal, require scaling approaches such as centered log-ratio normalization or quantile normalization that can preserve relative abundance across cells.

Another significant challenge lies in the computational scalability and infrastructure requirements. As multimodal platforms now routinely profile millions of cells across thousands of features, traditional computational pipelines often fail to manage the memory and processing demands of such large datasets [121]. Single-cell data’s inherent sparsity demands optimized algorithms capable of performing high-dimensional analyses at scale and memory-efficient storage formats, which reduces memory requirement by one to two orders of magnitude. Methods such as variational autoencoders (VAEs), graph neural networks, and other machine learning-based techniques often require high-performance computing (HPC) environments or graphics processing unit (GPU) acceleration due to the complexity of their deep neural network architecture, volume of data and the need for fast, large-scale matrix computations which exceed the capacity of typical central processing unit (CPU) for efficient training and inference [122]. When RNA and protein data are generated from different platforms such as 10x Genomics™ Chromium, BD Rhapsody™, or Parse Bioscience™ or across distinct laboratory batches or time points, platform-specific technical noise and batch effects are introduced. These sources of variability must be carefully addressed using robust harmonization techniques such as HarmonizR [123] and Harmony [124] to remove unwanted technical noise while retaining true biological signals.

A key computational challenge is modality alignment, which accurately matches each cell’s RNA and protein readouts despite data gaps. Dropout events, which are common in both modalities, often lead to partial observations where low-abundance features are undetected due to biological or technical factors. Although imputation methods, such as nearest-neighbour averaging or deep learning-based models, can estimate missing values, they risk introducing artifacts and obscuring true biological variation if not properly validated [125, 126]. Furthermore, mRNA-protein relationships are frequently nonlinear and context-dependent, rendering classical linear approaches such as canonical correlation analysis (CCA) inadequate for capturing complex regulatory mechanisms, as CCA models linear relationships between two sets of variables. This limitation has spurred demand for machine-learning frameworks capable of modelling nonlinear dependencies, which are an essential capability for accurately interpreting CITE-seq data and deriving biologically meaningful insights that linear methods cannot provide.

To address these computational challenges, a growing number of integrative analysis tools have been developed, each with unique strengths and limitations (Table 4). For instance, Seurat aligns modalities through canonical correlation analysis (CCA) coupled with mutual nearest-neighbors (MNN) matching, yet its reliance on linear models imposes limitations [127]. The Multi-Omics Factor Analysis Plus (MOFA+) uses factor analysis to identify shared and modality-specific sources of variation, yet performs suboptimally on extremely sparse datasets [128]. Total Variational Inference (TotalVI), a deep generative model built on the VAE framework, jointly denoises and imputes RNA and protein data while accounting for technical covariates, though its high computational cost limits scalability [126]. More recent approaches, such as the Single-cell Multi-omics Transformer-based Framework (scmFormer) [129], Single-cell Protein Reconstruction via Cross-Attention (scProca) [130], Single-cell Network-Enhanced Transformer (scNET) [131], and Single-cell Transformer Encoder for Labeling (scTEL) [132] utilize transformer architectures or graph neural networks for improved modelling of cross-modal relationships. While these methods offer improved performance and flexibility, they often come at the expense of interpretability, requiring careful benchmarking and biological validation. Table 4 summarizes the overview of the mentioned computational tools.

Looking forward, several key directions must be pursued to address the current limitations in multimodal data integration. First, algorithmic innovation is essential to develop methods that balance nonlinear modelling capacity with scalability and interpretability. Such innovation will enable the creation of integration frameworks that are not only accurate and scalable but also interpretable, capable of handling the multifaceted technical complexities inherent in modern single-cell multimodal datasets. Second, the field urgently needs standardized benchmarking frameworks to evaluate tool performance across diverse datasets and varying levels of technical noise, using biologically meaningful metrics. Without such standardized benchmarks, it becomes challenging to objectively compare methods, reproduce findings, or ensure methodological robustness in the face of highly variable and heterogeneous single-cell data. Finally, with the growing clinical adoption of single-cell technologies, transitioning from research settings into routine clinical applications for disease diagnosis, there is a growing need for robust, reproducible, and scalable pipelines. These pipelines must bridge the gap between complex single-cell assays and real-world clinical practice by delivering consistent, interpretable, and timely data to support personalized medicine. In particular, automated and clinically relevant pipelines that incorporate quality control, data integration, and biological interpretation are essential for reliably processing large volumes of diverse patient samples while maintaining consistent analytical quality and reproducibility. 

**Table 4:** Overview of computational tools for integrating single-cell transcriptomic and proteomic data

Tool	Modelling Approach	Integration Mode	Strengths	Limitations
Seurat (v4) [127]	Statistical integration using Canonical Correlation Analysis (CCA) and Weighted Nearest Neighbors (WNN)	Integrates RNA and ADT data; supports batch correction via anchors	• Widely used and well-documented • Flexible for various single-cell omics • Good batch correction	• Challenging multi-sample integration • Requires separate normalization for RNA and protein • Sensitive to batch effects and rare cell types
MOFA+ [128]	Bayesian factor analysis	Integrates matched multi-modal data; RNA and protein from same cells	• Unsupervised and interpretable factor discovery • Supports missing data • Differentiates shared and modality-specific variation	• Cannot model non-linear relationships • Assumes feature independence • Less effective at batch correction
TotalVI [126]	Deep generative model based on Variational Autoencoder (VAE)	Joint latent space for RNA and surface protein	• Denoises and integrates modalities simultaneously • Captures non-linear dependencies • Handles technical noise	• Requires GPU for efficient training • Difficulties in interpretating latent space • May be difficult for users without ML background
sciPENN [133]	Deep learning framework using repeated neural network blocks	Integrates CITE-seq and scRNA-seq data into a common latent embedding space	• Handles missing data using censored loss • Outperforms Seurat v4 and TotalVI in benchmarks • Provides uncertainty estimates for protein predictions • Robust to protocol and platform variability	• Requires high-quality matched RNA–protein reference datasets • Computationally intensive for large-scale training • Latent features may be less interpretable • May underperform in unseen biological contexts or rare cell types
scMHNN [134]	Hypergraph Neural Network with dual-contrastive self-supervised learning	Integrates tri-modal single-cell data (RNA, protein, epigenome) from same cells	• Captures high-order relationships across modalities • Effective with few labeled cells via self-supervised learning • Strong performance in clustering and annotation	• Requires matched tri-modal data • Computationally intensive • Complex architecture demands expertise to tune
scmFormer [129]	Transformer-based architecture with multi-task learning	Integrates scRNA-seq and CITE-seq/ASAP-seq data across batches and tissues; supports spatial datasets	• Scales to > 1.48 million cells on a personal computer • High accuracy in label transfer • Robust to batch effects • Can generate unmeasured modalities	• Requires significant resources for training • Complex attention mechanisms may obscure biological insights and interpretability
scProca [130]	Deep generative model combining VAE with cross-attention mechanisms.	Integrates scRNA-seq and CITE-seq, including partially overlapping ADT panels; can infer protein from RNA.	• Outperforms Seurat/TotalVI in integration metrics • Effectively removes batch effects while preserving biological variation • Supports large, multi-omics mosaic datasets	• Requires paired RNA-protein data for optimal performance • Complex training and tuning • Limited to ADTs, not full proteomes
scNET [131]	Dual-view Graph Neural Network (GNN) using gene–gene and cell–cell relationships	Integrates scRNA-seq with protein–protein interaction (PPI) networks	• Enhances identification of differentially enriched pathways and gene functional annotation. • Reduces noise and zero inflation typical of scRNA-seq data through graph-based smoothing.	• Focuses on integration of scRNA-seq with PPI networks rather than direct protein abundance measurements • Relies on availability and quality of PPI data • Model interpretability is limited due to GNN complexity
scTEL [132]	Deep learning framework using Transformer encoder layers	Predicts protein expression from scRNA-seq data, enabling joint analysis of RNA and protein in the same cell computationally	• Reduces experimental cost by computationally imputing protein expression instead of measuring it directly. • Integrates multiple CITE-seq datasets with partial protein overlap • Performs prediction, cell typing, and integration in one framework	• Relies on availability of high-quality paired RNA-protein training data • Prediction accuracy depends on the quality and representativeness of training data.

Abbreviation: *CCA* Canonical Correlation Analysis, *WNN* Weighted Nearest Neighbors, *ADT* Antibody-Derived Tag, *MOFA+* Multi-Omics Factor Analysis Plus, *TotalVI* Total Variational Inference, *sciPENN* Single Cell imputation Protein Embedding Neural Network, *VAE* Variational Autoencoder, *RNA* Ribonucleic Acid, *GPU* Graphics Processing Unit, *ML* Machine Learning, *scMHNN* Single-cell Multi-modal Hypergraph Neural Network, *scmFormer* Single-cell Multi-omics Transformer-based Framework, *CITE-seq* Cellular Indexing of Transcriptomes and Epitopes by Sequencing, *ASAP-seq* Assay for Single-cell Accessibility and Protein Sequencing, *scProca* Single-cell Protein Reconstruction via Cross-Attention, *scNET* Single-cell Network-Enhanced Transformer, *GNN* Graph Neural Network, *PPI* Protein–Protein Interaction, *scTEL* Single-cell Transformer Encoder for Labeling

## Current challenges and future directions

Despite the transformative potential of single-cell multi-omics in immunology, critical challenges persist in achieving precise immune cell annotation. First, it is still unclear whether multimodal single-cell data can fully resolve ambiguous intermediate immune states, which often display overlapping or transitional features. Second, limitations in antibody panels, such as incomplete coverage of emerging or rare markers, constrain the ability to accurately profile diverse immune populations at the protein level. Finally, zero-shot annotation of novel immune cell types remains a significant challenge, as current tools depend heavily on existing reference data and struggle to classify previously uncharacterized populations. Addressing these issues will be crucial for improving annotation accuracy and advancing our understanding of immune heterogeneity.

Additionally, current benchmarking remains difficult in part due to the absence of standardized metrics and comprehensive frameworks to reliably evaluate annotation accuracy across tools and datasets. Several web-based annotation platforms such as SCSA [135], Annotation of Cell Types (ACT) [136], CellHint [36] and curated resources like CellMarker database [137] have expanded accessibility for researchers by providing automated or semi-automated annotation pipelines. While these platforms accelerate cell-type identification, their accuracy remains dependent on the quality and completeness of reference marker sets or training datasets. As such, they may misclassify rare or novel immune populations or fail to capture subtle functional states, underscoring the continued need for well-curated, gold-standard reference datasets to ensure reliable benchmarking across methods.

Moreover, existing multimodal reference atlases primarily derive from healthy PBMCs or normal tissues, limiting their utility in disease contexts. Comprehensive atlases integrating matched mRNA, protein, and epigenomic data from tumor tissues remain scarce, hindering the development of computational models for precise annotation in cancer. While current integrative tools have advanced scalability and nonlinear modelling, they face limitations in interpretability and computational demands, often requiring GPUs and extensive training, which restricts their accessibility for routine analyses.

To address these gaps, future efforts may need to prioritize the development of a standardized, disease-inclusive multimodal atlas with matched molecular layers, coupled with community-driven benchmarks for tool validation. Scalable computational methods, such as lightweight neural networks or hybrid models integrating biological priors, must be optimized for usability without sacrificing interpretability. Collaborative initiatives, such as the Human Cell Atlas (HCA) [138], Single Cell Portal [139], scImmOmics [140] should expand to incorporate dynamic immune states such as activation or exhaustion, and clinical samples, enabling translation to therapeutic discovery and precision immunology.

## Data Availability

No datasets were generated or analysed during the current study.
